# Protocol for analyzing potential targets of environmental pollutants in human diseases using network toxicology and molecular docking

**DOI:** 10.1016/j.xpro.2026.104555

**Published:** 2026-05-11

**Authors:** Yanggang Hong, Jiajun Li, Jingxuan Zhou, Deqi Wang, Feng Chen, Yuze Mi, Yi Wang

**Affiliations:** 1 The Second Affiliated Hospital and Yuying Children’s Hospital of Wenzhou Medical University, Wenzhou, Zhejiang, China; 2 Wenzhou Medical University, Wenzhou, Zhejiang, China; 3 School of Urban Planning and Design, Peking University Shenzhen Graduate School, Shenzhen, China

**Keywords:** Gene Expression, Systems biology, Chemistry, Computer sciences, Environmental sciences

## Abstract

Here, we present a computational workflow for identifying and analyzing the molecular mechanisms through which environmental pollutants may contribute to human diseases. We describe steps for integrating network toxicology and molecular docking to enable systematic prediction of pollutant-target-disease relationships and structure-based plausibility assessment of molecular interactions. This protocol provides a reproducible and scalable framework applicable to diverse environmental compounds and disease models.

## Highlights

• Protocol for identifying shared pollutant-disease targets from public databases
• Workflow for PPI, module, hub gene, and functional enrichment analyses
• Guidance for structure-based docking of prioritized hub proteins
• Framework for hypothesis generation in toxicological mechanism studies

## Abstract (editor highlights)

Publisher’s note: Undertaking any experimental protocol requires adherence to local institutional guidelines for laboratory safety and ethics.

## Graphical abstract

**Figure:**
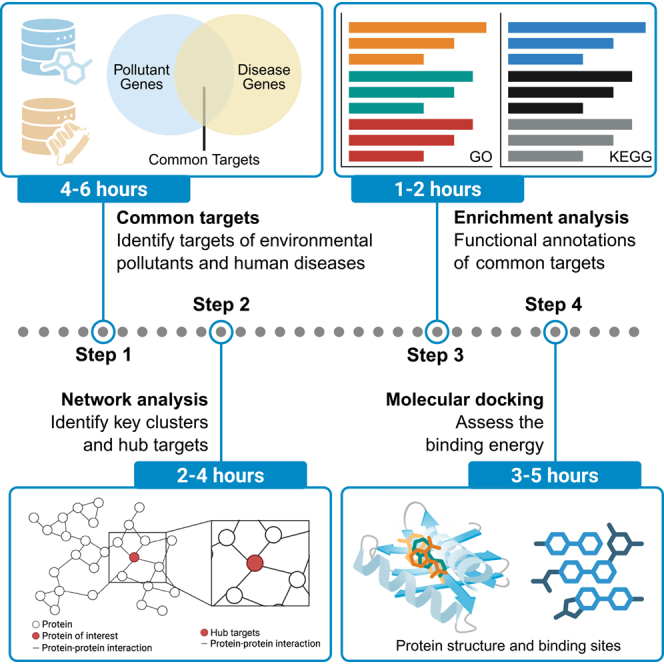

## Before you begin

### Overview

Environmental pollutants can disrupt normal cellular functions and contribute to disease onset through complex and multifactorial molecular interactions.^1^^,^^2^^,^^3^ Traditional toxicological studies often focus on single targets or pathways, which may overlook the broader network of biological processes affected by exposure to pollutants. To address this limitation, computational systems approaches such as network toxicology have emerged as powerful tools for integrating chemical, genomic, and disease information.^4^^,^^5^^,^^6^

This protocol provides a step-by-step workflow to systematically identify, analyze, and validate the potential molecular mechanisms underlying pollutant–disease associations.^7^ It integrates data from multiple public databases to construct interaction networks and applies molecular docking to verify the structural feasibility of key interactions. The approach facilitates the exploration of toxicity pathways, enables prediction of potential biomarkers, and supports hypothesis generation for experimental validation. This protocol offers a reliable and scalable framework for elucidating how environmental compounds may perturb human health at the molecular level.^8^

### Innovation

This protocol introduces an integrative and reproducible analytical framework that bridges large-scale bioinformatics with molecular modeling to study the biological impact of environmental pollutants. It advances traditional toxicology by shifting the focus from isolated molecular events to network-level interactions and structure-based validation. The integration of multi-database data mining, topological network analysis, and molecular docking provides a comprehensive understanding of pollutant–protein interactions and their potential disease relevance.

A major innovation of this workflow is its adaptability: the same analytical pipeline can be applied to any environmental compound or disease context with publicly available data. The standardized parameters, open-access databases, and widely used computational tools described here ensure reproducibility across research settings. By combining predictive network analysis with structure-based validation, this protocol serves as a versatile and scalable platform for mechanistic toxicology and environmental health research.^8^

### Database and software preparation

Before initiating the network toxicology and molecular docking workflow, ensure that all required databases and software tools are accessible and properly prepared. Stable internet access is required for querying public databases, and all computational tools should be installed and tested in advance to guarantee reproducibility and analytical stability.

1. Public databases, including PubChem, ChEMBL, STITCH, SwissTargetPrediction, GeneCards, OMIM, UniProt, STRING, and the RCSB Protein Data Bank, should be accessible, and the database access date and version should be recorded.
2. Cytoscape (v3.10.4) should be installed with the MCODE and CytoHubba plugins enabled for network construction and topological analysis.
3. R software (v4.3.2) should be installed with the clusterProfiler package and appropriate annotation libraries for functional enrichment analysis.
4. Molecular docking and structural visualization require PyMOL (v3.1), Molecular Operating Environment (MOE, 2019), ChemOffice (v20.0), and Discovery Studio Visualizer, all of which should be verified for proper operation prior to analysis.
5. A workstation with sufficient computational resources (recommended ≥16 GB RAM) is advised to ensure smooth execution of network analyses and docking simulations.

***Note:*** Equivalent databases or software tools may be used as alternatives, provided that software versions, parameters, and database access dates are clearly documented.

## Key resources table

**Table:** 

REAGENT or RESOURCE	SOURCE	IDENTIFIER
**Software and algorithms**		

Cytoscape v.3.10.4	Shannon et al., 2003^9^	https://cytoscape.org
MCODE	Bader et al., 2003^10^	https://apps.cytoscape.org/apps/mcode
CytoHubba	Chin et al., 2014^11^	https://apps.cytoscape.org/apps/cytohubba
R software v.4.3.2	The R Foundation	https://www.r-project.org
clusterProfiler v.4.14.6	Wu et al., 2021^12^	https://github.com/YuLab-SMU/clusterProfiler
PyMOL v.3.1	Schrödinger, LLC	https://pymol.org
ChemOffice (v20.0)	Revvity Signals	https://revvitysignals.com/products/research/chemdraw
MOE (2019)	Chemical Computing Group Inc.	https://www.chemcomp.com/Products.htm
Discovery Studio Visualizer	Dassault Systèmes BIOVIA	https://www.3ds.com/products/biovia/discovery-studio/visualization

**Other**		

PubChem	Kim et al., 2025^13^	https://pubchem.ncbi.nlm.nih.gov
ChEMBL	Zdrazil et al., 2024^14^	https://www.ebi.ac.uk/chembl/
STITCH	Szklarczyk et al., 2016^15^	http://stitch.embl.de
SwissTargetPrediction	Daina et al., 2019^16^	http://www.swisstargetprediction.ch
GeneCards	Safran et al., 2010^17^	https://www.genecards.org
OMIM	Amberger et al., 2019^18^	https://omim.org
UniProt	UniProt Consortium, 2025^19^	https://www.uniprot.org
STRING	Szklarczyk et al., 2025^20^	https://string-db.org
PDB	Berman et al., 2000^21^	https://www.rcsb.org

## Step-by-step method details

This section describes the complete computational workflow for identifying and validating the potential molecular targets of environmental pollutants in human diseases. The process integrates multi-database target collection, network construction, enrichment analysis, and molecular docking validation. Each step is modular and can be adapted for different compounds or disease models. In the present protocol, bisphenol A (BPA) and osteosarcoma are used as a representative worked example to illustrate the implementation of the workflow.

### Compilation of pollutant-associated targets

**Timing: 2–3 h**

**Figure 1:**
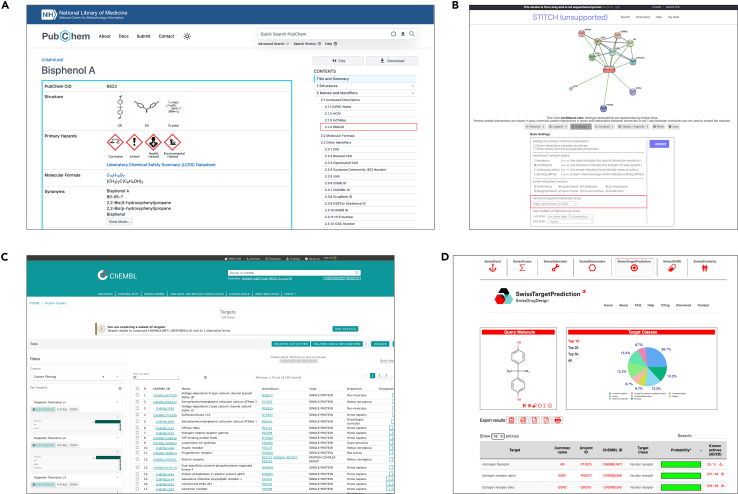
Visual guidance for compiling pollutant-associated targets from public databases Representative screenshots illustrating the key database operations used in the worked example for BPA. (A) PubChem compound record used to retrieve chemical identifiers and structural information. (B) STITCH interface used to set the organism and interaction score threshold for exporting chemical–protein interactions. (C) ChEMBL target/bioactivity results page used to collect human pollutant-associated targets. (D) SwissTargetPrediction output page used to identify predicted human targets from the canonical SMILES input. These screenshots are included to provide step-by-step visual guidance and to help readers verify that they are following the protocol correctly.
  b. Copy and record the following into 01_Targets/pollutant_metadata.txt: PubChem CID, Canonical SMILES, InChIKey, and isomeric SMILES.
3. Query pollutant-associated targets using public chemical and bioactivity databases.
  a. STITCH^15^ query (Figure 1B):
    i. Search the compound and open the corresponding result page.
    ii. Set the organism to *Homo sapiens*; open the Settings panel.
    iii. Under minimum required interaction score, select high confidence (0.700).
    iv. Click Update.
    v. Export the chemical–protein interactions from Tables/Exports.
    vi. Save the file as 01_Targets/STITCH_<Pollutant>_raw.tsv.
  b. ChEMBL^14^ query (Figure 1C):
    i. Search by SMILES (preferred) or CID/name.
    ii. Open the compound record; navigate to the target/bioactivity section.
    iii. Set the organism filter to *Homo sapiens*; retain targets with reported bioactivity records (Activities > 0).
    iv. Export the target/bioactivity table.
    v. Save the file as 01_Targets/ChEMBL_<Pollutant>_raw.csv.
  c. SwissTargetPrediction^16^ query (Figure 1D):
    i. Paste the canonical SMILES.
    ii. Retain predicted targets with probability > 0.
    iii. Export the predicted target table.
    iv. Save the file as 01_Targets/SwissTargetPrediction_<Pollutant>_raw.csv.
  d. Save a simple per-source summary that records the number of targets retrieved from each source in 01_Targets/pollutant_target_source_summary.txt such as:
    i. “STITCH: *N* =__ targets”.
    ii. “ChEMBL: *N* =__ targets”.
    iii. “SwissTargetPrediction: *N* =__ predicted targets”.
4. Merge, standardize, and de-duplicate all retrieved targets using UniProt ID mapping.^19^
  a. Combine the three raw exports into a single spreadsheet/table 1_Targets/pollutant_merged_raw_targets.xlsx with columns: Source, Original_ID, Protein_name, Organism, Evidence/Score, Notes.
  b. Upload all identifiers to UniProt ID mapping: map to HGNC gene symbol and UniProtKB accession.
  c. Remove duplicates using the HGNC gene symbol as the primary key; when duplicates occur across sources, keep a column Sources_supporting (e.g., “STITCH; ChEMBL; SwissTargetPrediction”).
  d. Flag ambiguous mappings:
    i. One identifier mapping to multiple genes.
    ii. Obsolete symbols.
    iii. Non-human proteins.
  e. Save flagged entries in 01_Targets/pollutant_mapping_qc_flags.csv and exclude them unless manually resolved.
  f. Output the final normalized list as:
    i. 01_Targets/pollutant_targets_all.txt (one official gene symbol per line).
    ii. 01_Targets/pollutant_targets_all.csv (detailed table with sources/evidence).

***Note:*** For multiple-pollutant exposure analyses, this workflow can be adapted by first generating pollutant-specific target lists for each compound, and then deriving either a union set or an intersection set, depending on whether the aim is to investigate cumulative effects or shared mechanisms.^8^

1. Define the pollutant and create the project folder.
  a. Define the pollutant clearly (common name + synonym) and create a project folder (Project_Pollutant_Disease/01_Targets/).
  b. In a text file 01_Targets/pollutant_metadata.txt, record: pollutant name and synonyms.
2. Retrieve chemical identifiers from PubChem database.^13^
  a. Search the pollutant in the PubChem database and select the correct compound page (Figure 1A).

**CRITICAL:** Ensure that the compound’s structural representation (SMILES or InChI) is correct and consistent across databases to prevent mismatched targets.

### Identification of disease-associated targets

**Timing: 1–2 h**

**Figure 2:**
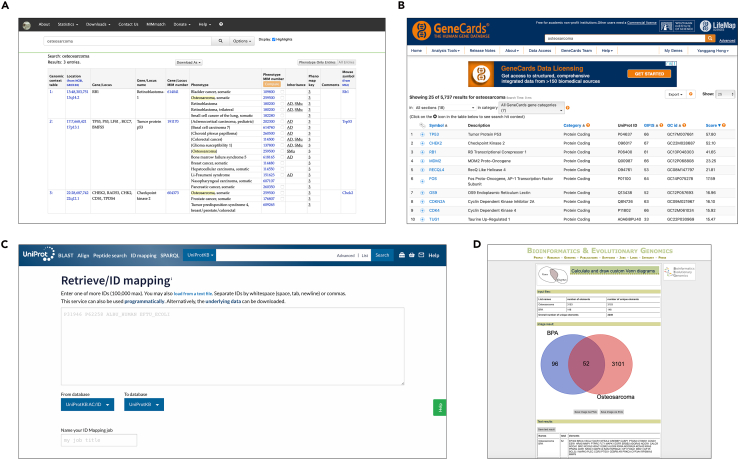
Visual guidance for identifying disease-associated genes and generating the overlap set Representative screenshots illustrating the key database operations used in the worked example for osteosarcoma. (A) OMIM search results page used to retrieve disease-associated genes from phenotype-relevant entries. (B) GeneCards results page showing osteosarcoma-related genes and the associated relevance score information used for filtering. (C) UniProt ID mapping interface used for harmonizing disease gene identifiers. (D) Venn diagram output used to visualize the intersection between pollutant-associated targets and disease-associated genes. These screenshots serve as inline visual checkpoints to support reproducible execution of the protocol.
  b. GeneCards retrieval (Figure 2B)
    i. Open GeneCards, paste the disease keyword, and enter the disease results page.
    ii. Navigate to the “Genes” section and export/download the full gene list.
    iii. Save as 01_Targets/GeneCards_<Disease>_raw.csv.
  c. Record in disease_metadata.txt: the OMIM phenotype entry ID(s) used (if multiple, list them all), and query date.
  d. Ensure GeneCards and OMIM outputs include gene identifiers that can be mapped (symbols/UniProt/Entrez); if not, note the ID type.
7. Filter the GeneCards results by relevance score.^17^
  a. Open GeneCards_<Disease>_raw.csv and confirm the presence of a relevance score column (or equivalent).
  b. Compute the median relevance score from the exported table (do not eyeball). Record the value in disease_metadata.txt as: “GeneCards median relevance score (run date YYYY-MM-DD) = ___”.
  c. Filter rule: retain genes with score ≥ median.
  d. Save the filtered GeneCards list as:
    i. 01_Targets/GeneCards_<Disease>_filtered.txt (one gene symbol per line).
    ii. 01_Targets/GeneCards_<Disease>_filtered.csv (retain score column for transparency).
  e. Report counts in the text: “GeneCards returned *N* = __ genes; median score = __; retained *N* = __ genes after filtering.”

***Note:*** The median relevance score is used as the default threshold to retain highly associated genes. Alternative thresholds may also be applied depending on the study objective, but the selected criterion should be stated explicitly and justified appropriately.

5. Search for disease-related genes in GeneCards and OMIM databases.^17^^,^^18^
  a. Define the disease keyword(s) (preferred: official disease name + common synonym). Create a record file: 01_Targets/disease_metadata.txt.
  b. In disease_metadata.txt, record: disease name(s), the exact search keyword string, query date/time, and database URLs.
  c. Create a working folder: Project_Pollutant_Disease/01_Targets/, and keep raw exports separately for GeneCards and OMIM.
6. Use the disease name as the keyword and retrieve all associated genes.
  a. OMIM retrieval (Figure 2A)
    i. Open OMIM, search the disease keyword, and open the most relevant phenotype entry.
    ii. Copy genes listed in “Gene(s)”/“Phenotype–Gene Relationships” into a text file.
    iii. Save as 01_Targets/OMIM_<Disease>_genes_raw.txt.

8. Combine the results from both databases, remove duplicates, and convert all gene identifiers to official gene symbols using UniProt.^19^
  a. Merge GeneCards_<Disease>_filtered with OMIM_<Disease>_genes_raw into a single table 01_Targets/disease_genes_merged_raw.xlsx with columns: Source, Original_ID, Gene_symbol_if_any, Notes.
  b. Use UniProt ID mapping to standardize all identifiers to official HGNC gene symbols and (optionally) UniProt accessions (Figure 2C).
  c. Remove duplicates using the HGNC gene symbol as the primary key; keep a column Sources_supporting (e.g., “GeneCards;OMIM”) for each gene.
  d. Save final disease-associated gene list as:
    i. 01_Targets/disease_genes_final.txt (one official gene symbol per line).
    ii. 01_Targets/disease_genes_final.csv (detailed table with sources and mapping).
  e. Create a QC file for mapping failures and manual checks: 01_Targets/disease_mapping_qc_flags.csv containing:
    i. Unmapped IDs.
    ii. Multi-mapped IDs.
    iii. Non-human hits if present.
  f. Report final size: “The final disease-associated gene set contained *N* = __ genes.”

***Note:*** Adjust the threshold according to the disease type and dataset size.

**Pause point:** The curated disease-associated gene list can be saved for subsequent analyses.

### Identification of common targets between pollutants and disease

**Timing: 30–45 min**

9. Compare the pollutant- and disease-associated target lists to identify common genes.
  a. Ensure both input files are plain text with one official HGNC gene symbol per line (no header).
    i. 01_Targets/pollutant_targets_all.txt.
    ii. 01_Targets/disease_genes_final.txt.
  b. Compute intersection and export the overlap list as: 02_Intersection/overlap_genes.txt (one gene symbol per line).
  c. Report the overlap size in the text: “The overlap contained *N* = __ genes.”

***Note:*** These common targets represent potential molecular links between pollutant exposure and disease pathogenesis.

10. Use an online tool for Venn diagram or in-house scripts to visualize the intersection.
  a. Paste the gene lists (pollutant_targets_all.txt and disease_genes_final.txt) into a Venn diagram web tool (http://bioinformatics.psb.ugent.be/webtools/Venn/).
  b. Generate the Venn diagram and export the image as: 02_Intersection/Figure 2_Venn.svg (Figure 2D).
  c. Export/download the overlap list if the tool supports it; otherwise, manually copy the overlap genes and save as 02_Intersection/overlap_genes.txt.

**CRITICAL:** Double-check for consistent naming and identifier formats before intersection; mismatched gene names may result in loss of overlapping entries.

### Construction and analysis of the PPI network

**Timing: 2–4 h**

**Figure 3:**
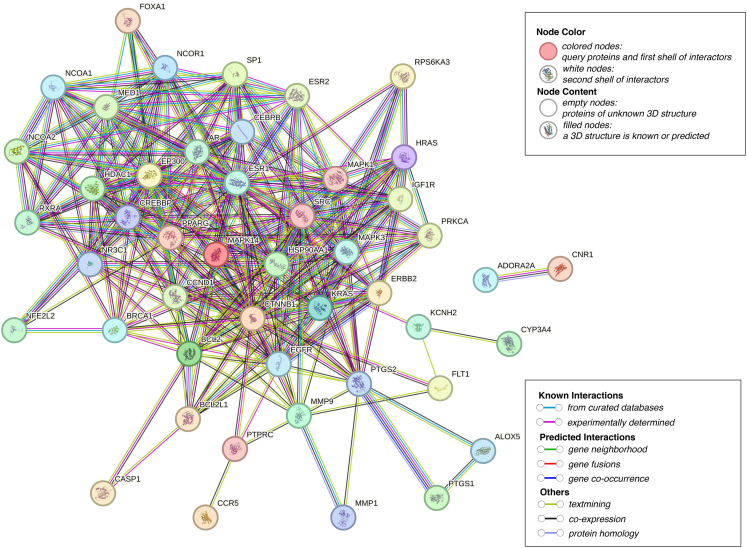
PPI network generated using the STRING database of common targets The common targets were imported into the STRING database to construct a PPI network. Nodes represent common targets, and edges represent known or predicted functional associations between proteins. The network shows extensive interconnectivity among the common targets, suggesting that these proteins may participate in coordinated biological processes relevant to the potential effects of BPA on osteosarcoma.
  f. Export the interaction table as 03_PPI_STRING/string_interactions.tsv.
  g. Visually confirm the network is not excessively sparse (too few edges) or overly dense (hairball).

***Note:*** A minimum required interaction score of 0.700 is recommended to balance network reliability and interpretability. Alternative thresholds may be used depending on the study purpose and desired network stringency, but the selected criterion should be explicitly stated and justified.

11. Import the overlapping targets into the STRING database to construct a protein–protein interaction (PPI) network.^20^
  a. Prepare the input file 02_Intersection/overlap_genes.txt containing one official HGNC gene symbol per line (no commas/semicolons).
  b. Open STRING database, and choose “Multiple proteins”.
  c. Paste the overlap gene symbols into the input box (or upload the .txt file).
  d. Select Organism = *Homo sapiens* before submitting the query.
  e. After STRING maps identifiers, download/save the mapping report to 03_PPI_STRING/string_mapping_report.tsv.
  f. Confirm that the number of mapped proteins is close to the number of input genes.
12. Set the species to *Homo sapiens* and define the STRING confidence score threshold.
  a. On the STRING network page, set:
    i. Set the meaning of network edges to “evidence” with default active interaction sources used.
    ii. Minimum required interaction score: set to 0.700 (high confidence).
  b. Specify whether disconnected nodes are shown/hidden (recommended: hide disconnected nodes for clarity, but keep them recorded).
  c. Specify which active interaction sources are included (e.g., experiments, databases, co-expression, textmining). If keeping defaults, write “default interaction sources in STRING were used.”
  d. Record these settings in 03_PPI_STRING/string_settings.txt (including query date).
  e. Export an image of the STRING network for documentation: 03_PPI_STRING/Figure 3_STRING_network.svg (Figure 3).

13. Export the interaction data and import it into Cytoscape for visualization and further analysis.^9^
  a. In STRING, export network data as tabular files:
    i. Edges/links table: save as 03_PPI_STRING/string_edges.tsv
    ii. Nodes table: save as 03_PPI_STRING/string_nodes.tsv
  b. Open Cytoscape (v3.10.4) > File > Import > Network from File > select string_interactions.tsv.
  c. If needed, import node attributes: File > Import > Table from File > select string_nodes.tsv and map by protein/gene identifier column.
  d. Save Cytoscape session immediately: 04_Cytoscape/PPI_overlap_STRING.cys.
  e. Export an image of the Cytoscape network for documentation: 03_PPI_STRING/Figure 4_Cytoscape_network.svg (Figure 4).

**Figure 4:**
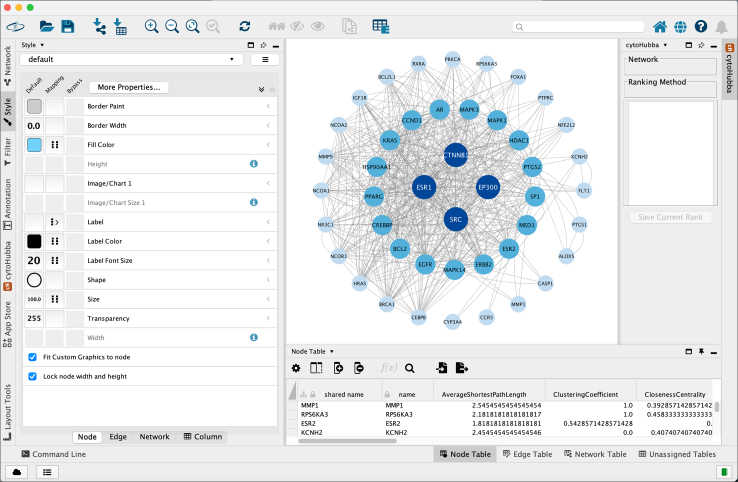
Network visualization of common targets ranked by degree centrality in Cytoscape The common targets were ranked according to their degree values in the PPI network to identify highly connected genes. In this network, each node represents a common target and each edge represents a PPI. Node size and color intensity both reflect the degree value, with larger and darker blue nodes indicating higher connectivity within the network. Genes such as ESR1, SRC, EP300, and CTNNB1 showed the highest degree values and occupied central positions, suggesting that they may function as key regulatory targets linking BPA to osteosarcoma.
  f. Confirm the node count and edge count in Cytoscape match the STRING export (± minor differences due to filtering).
14. Apply the Molecular Complex Detection (MCODE) plugin to identify network clusters.^10^
  a. In Cytoscape, open Apps (MCODE), and start MCODE.
  b. Use and report the exact parameters:
    i. Degree cutoff = __ (commonly 2)
    ii. Node score cutoff = __ (commonly 0.2).
    iii. K-core = __ (commonly 2).
    iv. Max depth = __ (commonly 100).
  c. Click “Analyze” to detect clusters/modules.
  d. Export results:
    i. Cluster summary table: 04_Cytoscape/MCODE_cluster_summary.csv.
    ii. For each top cluster, export member genes: 04_Cytoscape/MCODE_cluster1_genes.txt, cluster2_genes.txt, etc.
  e. Generate and export the module visualization as: 04_Cytoscape/Figure 5_MCODE_modules.svg (Figure 5).

**Figure 5:**
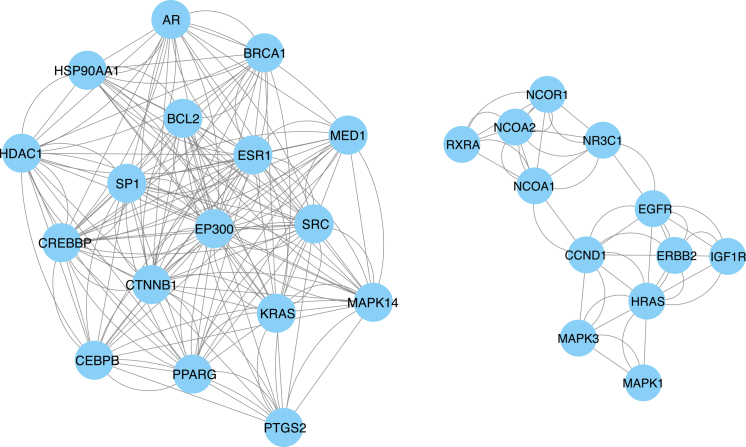
Modules of functionally related proteins identified using MCODE in Cytoscape Subnetwork analysis of the common target interaction network revealed two relatively distinct but internally connected modules. The left module shows a densely interconnected cluster centered around genes such as ESR1, EP300, CTNNB1, BCL2, SRC, and KRAS, whereas the right module contains another connected group including EGFR, HRAS, ERBB2, IGF1R, MAPK1, MAPK3, NR3C1, NCOR1, NCOA1, and NCOA2. These modules suggest that the common targets are organized into functionally related subnetworks rather than forming a completely uniform interaction structure. This step helps refine the overall PPI network into interpretable clusters and supports the identification of biologically meaningful target groups for downstream functional analysis and molecular docking.
  f. Record the number of clusters and the size of the top cluster (e.g., “Top cluster contained __ nodes”).
15. Use the CytoHubba plugin to analyze network topology and identify hub proteins based on metrics like maximal clique centrality (MCC), edge percolated component (EPC), closeness, and betweenness.^11^
  a. In Cytoscape, open Apps > cytoHubba.
  b. Define a hub selection rule before running (must be explicit for reproducibility):
    i. Primary metric: MCC (recommended).
    ii. Top *N* hubs: Top 10 (or Top 20).
  c. Run cytoHubba with MCC, set Top *N* = __, and export ranking: 04_Cytoscape/cytoHubba_MCC_TopN.csv
  d. Repeat for EPC, Closeness, and Betweenness with the same Top N, export each:
    i. cytoHubba_EPC_TopN.csv.
    ii. cytoHubba_Closeness_TopN.csv.
    iii. cytoHubba_Betweenness_TopN.csv.
  e. Create a consensus hub list: Intersection of the four Top N lists and save as 04_Cytoscape/hubs_consensus_intersection.txt.
  f. Export Figure 6 panels (A–D) as high-resolution images:
    i. 04_Cytoscape/Figure 6A_EPC.svg (Figure 6A).
    ii. Figure 6B_Closeness.svg (Figure 6B).
    iii. Figure 6C_MCC.svg (Figure 6C).
    iv. Figure 6D_Betweenness.svg (Figure 6D).

**Figure 6:**
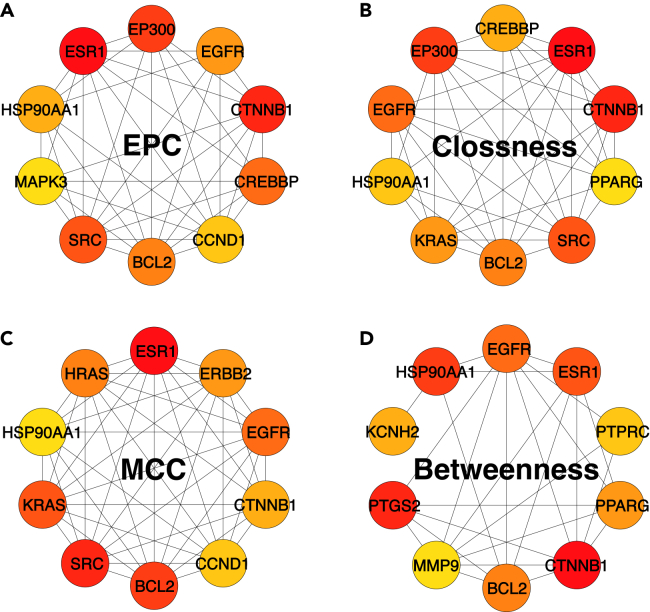
Identification of hub targets based on different topological parameters Hub genes within the common BPA–osteosarcoma target network were ranked using four topological methods in cytoHubba: (A) EPC, (B) Closeness, (C) MCC, and (D) Betweenness. Each panel shows the top-ranked genes identified by the corresponding algorithm, with warmer node colors indicating higher topological importance within that ranking method. Several genes, including BCL2, CTNNB1, EGFR, ESR1, and HSP90AA1, were repeatedly identified across multiple algorithms, suggesting that they may represent robust hub genes in the common target network. This multi-algorithm comparison was used to improve confidence in hub gene prioritization and to guide subsequent functional analysis and molecular docking.
  g. Save the final hub list that will be used for molecular docking as: 04_Cytoscape/hub_targets_final.txt (one gene per line).
  h. Report the Top hubs identified.

***Note:*** When relevant datasets are available, this workflow can be further strengthened by integrating tissue- or cell-type-relevant expression data, such as differentially expressed genes, tissue-enriched genes, or single-cell markers, to refine hub gene prioritization and improve the biological specificity of downstream functional analyses.^6^

**CRITICAL:** Avoid including isolated nodes or low-confidence edges, as they may introduce noise in downstream analyses.

### Functional enrichment analysis

**Timing: 1–2 h**

16. Perform GO and KEGG enrichment analyses for the common targets.
  a. Prepare the overlap gene list as the enrichment input (choose ONE and state it clearly): 02_Intersection/overlap_genes.txt.
  b. Save the final input list used for enrichment as: 05_Enrichment/enrichment_input_genes.txt (one official HGNC symbol per line).
  c. Create an output folder: 05_Enrichment/, and keep all intermediate files.
  d. Decide the universe/background gene set (must be consistent across GO/KEGG):
    i. Default option: all Entrez genes in org.Hs.e.g.db (state “default background in clusterProfiler”).
    ii. Save as 05_Enrichment/background_genes.txt.
  e. Record input gene count (*N*) and background gene count (*M*) in 05_Enrichment/enrichment_metadata.txt.

***Note:*** These analyses are used to identify enriched biological processes, cellular components, molecular functions, and pathways associated with the common targets.

**Figure 7:**
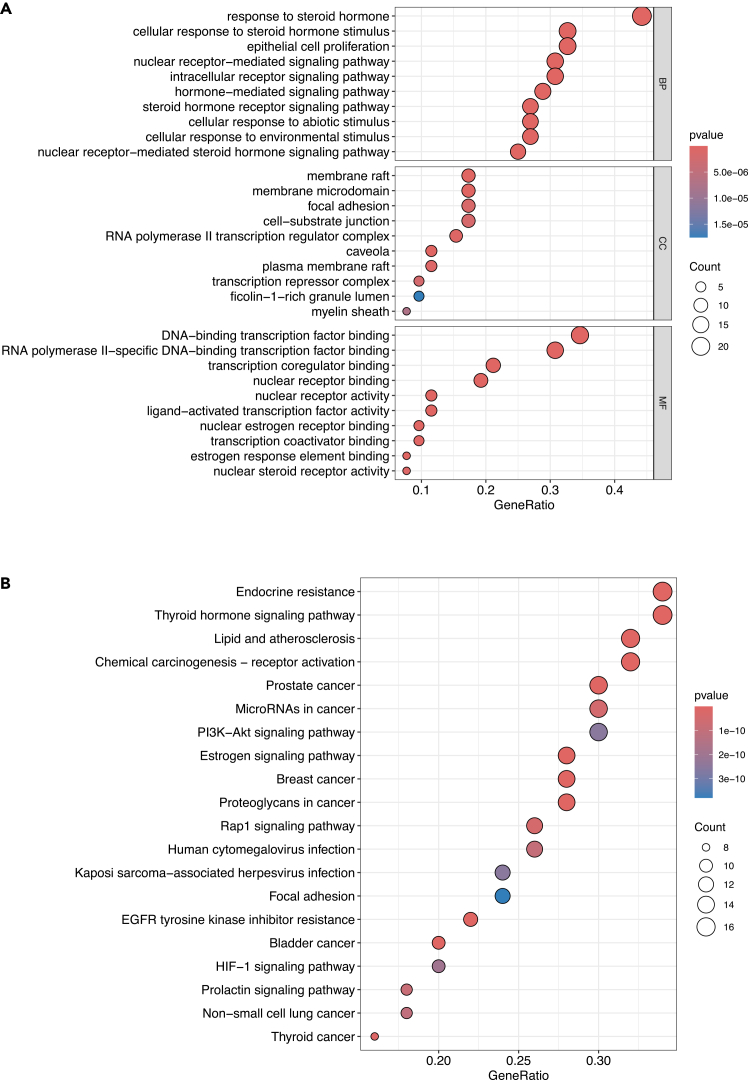
Enrichment analyses of common targets between the selected environmental pollutant and the selected disease (A) GO enrichment analysis of the common targets, including BP, CC, and MF categories. The enriched GO terms were mainly related to steroid hormone response, nuclear receptor-mediated signaling, epithelial cell proliferation, membrane raft/microdomain organization, focal adhesion, and transcription regulator binding, suggesting that the common targets are involved in hormone-responsive signaling and transcriptional regulation. (B) KEGG pathway enrichment analysis of the same target set. The significantly enriched pathways included endocrine resistance, thyroid hormone signaling pathway, estrogen signaling pathway, PI3K–Akt signaling pathway, focal adhesion, and multiple cancer-related pathways. These enrichment results indicate that the common targets may participate in hormone-related and oncogenic signaling processes relevant to the potential effects of BPA on osteosarcoma, and they provide a functional basis for hub gene interpretation and molecular docking.
  c. Generate KEGG plots: Dot plot: 05_Enrichment/Figure 7B_KEGG_dotplot.pdf (Figure 7B).
  d. Save the exact R script used as 05_Enrichment/enrichment_clusterProfiler.R and save sessionInfo() output to 05_Enrichment/sessionInfo.txt.

17. Use the clusterProfiler package in R for the analysis, applying the Benjamini–Hochberg (BH) adjusted *p*-value cutoff of <0.05 to define significant enrichment.^12^
  a. Start R Studio and load packages: clusterProfiler, org.Hs.e.g.db, enrichplot, ggplot2, dplyr.
  b. Read 05_Enrichment/enrichment_input_genes.txt as gene symbols and convert to ENTREZID using bitr() (recommended) or AnnotationDbi::select().
  c. Save the mapping table for transparency: 05_Enrichment/gene_symbol_to_entrez.csv, including unmapped symbols.
  d. Define enrichment parameters:
    i. pAdjustMethod = “BH”.
    ii. pvalueCutoff = 0.05.
    iii. qvalueCutoff = 0.05.
    iv. minGSSize = 10, maxGSSize = 500.
    v. readable = TRUE (for gene symbols in output).
  e. Run GO enrichment separately for BP/CC/MF: enrichGO(ont=“BP”), enrichGO(ont=“CC”), enrichGO(ont=“MF”).
  f. Run KEGG enrichment: enrichKEGG(organism=“hsa”) (or enrichKEGG(gene = entrez, organism = “hsa”)).
  g. Export all enrichment result tables as CSV:
    i. 05_Enrichment/GO_BP_results.csv.
    ii. 05_Enrichment/GO_CC_results.csv.
    iii. 05_Enrichment/GO_MF_results.csv.
    iv. 05_Enrichment/KEGG_results.csv.
  h. Confirm that significant terms exist (BH-adjusted *p*-value < 0.05).
18. Visualize the results using bar or bubble plots to highlight the most enriched terms and pathways.
  a. Decide the number of top terms to plot: Top 10 terms by adjusted *p*-value for each ontology/pathway.
  b. Generate GO plots: Dot plot: 05_Enrichment/Figure 7A_GO_dotplot.pdf (Figure 7A).

library(clusterProfiler)

library(org.Hs.eg.db)

library(enrichplot)

library(ggplot2)

library(dplyr)

library(AnnotationDbi)

# Read input genes

genes <- readLines("05_Enrichment/enrichment_input_genes.txt")

# Define background genes

bg <- keys(org.Hs.eg.db, keytype = "ENTREZID")

writeLines(bg, "05_Enrichment/background_genes.txt")

# Convert gene symbols to ENTREZ IDs

map <- bitr(genes,

   fromType = "SYMBOL",

   toType = "ENTREZID",

   OrgDb = org.Hs.eg.db)

if (nrow(map) == 0) stop("No gene symbols could be mapped to ENTREZID.")

map_all <- data.frame(SYMBOL = genes) %>%

left_join(map, by = "SYMBOL")

write.csv(map_all, "05_Enrichment/gene_symbol_to_entrez.csv", row.names = FALSE)

entrez <- unique(na.omit(map$ENTREZID))

# Save enrichment metadata

writeLines(c(

paste0("Input gene count (N): ", length(genes)),

paste0("Background gene count (M): ", length(bg))

), "05_Enrichment/enrichment_metadata.txt")

# Run GO enrichment for BP

ego_bp <- enrichGO(gene = entrez, OrgDb = org.Hs.eg.db, keyType = "ENTREZID",

   ont = "BP", universe = bg,

   pAdjustMethod = "BH", pvalueCutoff = 0.05, qvalueCutoff = 0.05,

   minGSSize = 10, maxGSSize = 500, readable = TRUE)

# Run GO enrichment for CC

ego_cc <- enrichGO(gene = entrez, OrgDb = org.Hs.eg.db, keyType = "ENTREZID",

   ont = "CC", universe = bg,

   pAdjustMethod = "BH", pvalueCutoff = 0.05, qvalueCutoff = 0.05,

   minGSSize = 10, maxGSSize = 500, readable = TRUE)

# Run GO enrichment for M F

ego_mf <- enrichGO(gene = entrez, OrgDb = org.Hs.eg.db, keyType = "ENTREZID",

   ont = "MF", universe = bg,

   pAdjustMethod = "BH", pvalueCutoff = 0.05, qvalueCutoff = 0.05,

   minGSSize = 10, maxGSSize = 500, readable = TRUE)

# Run KEGG enrichment

ekegg <- enrichKEGG(gene = entrez, organism = "hsa", universe = bg,

   pAdjustMethod = "BH", pvalueCutoff = 0.05, qvalueCutoff = 0.05,

   minGSSize = 10, maxGSSize = 500)

# Export enrichment results

write.csv(as.data.frame(ego_bp), "05_Enrichment/GO_BP_results.csv", row.names = FALSE)

write.csv(as.data.frame(ego_cc), "05_Enrichment/GO_CC_results.csv", row.names = FALSE)

write.csv(as.data.frame(ego_mf), "05_Enrichment/GO_MF_results.csv", row.names = FALSE)

write.csv(as.data.frame(ekegg), "05_Enrichment/KEGG_results.csv", row.names = FALSE)

# Generate GO dot plot (BP shown as example)

pdf("05_Enrichment/ Figure7A _GO_dotplot.pdf", width = 8, height = 6)

print(dotplot(ego_bp, showCategory = 10))

dev.off()

# Generate KEGG dot plot

pdf("05_Enrichment/ Figure7B _KEGG_dotplot.pdf", width = 8, height = 12)

print(dotplot(ekegg, showCategory = 20))

dev.off()

# Save session information

writeLines(capture.output(sessionInfo()), "05_Enrichment/sessionInfo.txt")

***Note:*** Use consistent background gene sets to ensure comparability between analyses.

**CRITICAL:** Double-check gene identifiers (ENTREZ ID or gene symbol) before analysis to avoid annotation errors.

### Molecular docking

**Timing: 3–5 h**

19. Obtain the 3D structure of the small-molecule ligand from the PubChem database and save it in SDF or MOL2 format.
  a. Open PubChem and search the pollutant.
  b. Record PubChem identifiers in 06_Docking/ligand_metadata.txt: compound name, CID, canonical SMILES, InChIKey, download date.
  c. In PubChem, download the 3D conformer in SDF format and save as: 06_Docking/ligand_raw.sdf.
  d. If MOL2 is required downstream, convert SDF to MOL2 later in MOE or ChemOffice; keep the raw SDF unchanged for traceability.
20. Optimize the ligand structure using ChemOffice 20.0 to perform geometry optimization and energy minimization.
  a. Open ligand_raw.sdf in ChemOffice (Chem3D).
  b. Perform geometry optimization/energy minimization using default or specified force field.
  c. Save the minimized ligand as: 06_Docking/ligand_min.mol2 and/or 06_Docking/ligand_min.sdf.
  d. Confirm the ligand has correct protonation/valence and no broken bonds; if ChemOffice alters stereochemistry, revert and re-import the original SDF.
21. Download the high-resolution crystal structure of the target protein receptor from the RCSB Protein Data Bank (PDB).
  a. Select receptor targets.
  b. For each protein, define a PDB selection rule and record it in 06_Docking/receptor_selection_rule.txt:
    i. Method: X-ray crystallography or cryo-EM.
    ii. Resolution cutoff (recommended): ≤ 2.5 Å.
    iii. Prefer ligand-bound structures to define the active pocket.
  c. Download the chosen PDB file(s) and save as:
    i. 06_Docking/BCL2_<PDBID>.pdb.
    ii. 06_Docking/CTNNB1_<PDBID>.pdb.
    iii. 06_Docking/EGFR_<PDBID>.pdb.
    iv. 06_Docking/ESR1_<PDBID>.pdb.
    v. 06_Docking/HSP90AA1_<PDBID>.pdb.
  d. Record for each receptor in 06_Docking/receptor_metadata.csv: Gene, PDB ID, resolution, chain used, presence of co-crystal ligand, download date.
22. Prepare the protein receptor using PyMOL 3.1 by removing water molecules, ions, and any co-crystallized ligands.
  a. Open each receptor PDB in PyMOL.
  b. Remove non-essential molecules:
    i. Delete waters (HOH), salts/ions, buffer molecules.
    ii. Remove co-crystallized ligand only if not using it for pocket definition or redocking.
  c. Keep the biologically relevant chain(s) and remove unrelated chains if present; record the chain ID(s) retained.
  d. Save the cleaned receptor as: 06_Docking/<Protein>_<PDBID>_clean.pdb.
  e. Ensure the receptor file contains standard residues only (no missing atoms warnings if possible); if the binding site is incomplete, choose an alternative PDB.

***Note:*** The receptor preparation operations may be described in text rather than as explicit software commands.

23. Import the prepared ligand and receptor structures into Molecular Operating Environment (MOE) 2019.
  a. Launch MOE 2019 and create a dedicated project directory 06_Docking/MOE_project/.
  b. Import ligand_min.mol2 and <Protein>_<PDBID>_clean.pdb into MOE.
  c. Save the MOE database/session file: 06_Docking/MOE_project/<Protein>_<PDBID>.mdb.
  d. Confirm both structures display correctly and the ligand is recognized as a small molecule (not merged into protein).
24. Perform ligand energy minimization and identify the active binding pocket of the receptor within MOE.
  a. Ligand minimization in MOE: run energy minimization again inside MOE (to ensure compatibility with MOE atom typing). Save as 06_Docking/ligand_MOEmin.mol2.
  b. Receptor preparation: add hydrogens and assign protonation/partial charges using MOE’s protein preparation workflow; record settings in 06_Docking/MOE_settings.txt.
  c. Binding pocket definition: define pocket based on co-crystallized ligand location (active site).
  d. Save pocket/site information or at minimum record: pocket selection method, residues included, and rationale in MOE_settings.txt.
  e. Visually confirm the pocket encloses key residues and is not placed on an exposed irrelevant surface.
25. Conduct molecular docking simulations using MOE.
  a. Open MOE Dock panel and set iteration number = 200-1,000.
  b. Specify and record the full docking configuration in 06_Docking/MOE_settings.txt:
    i. Placement method (e.g., Triangle Matcher).
    ii. Initial scoring function (e.g., London dG).
    iii. Refinement method (e.g., Induced Fit/Rigid receptor).
    iv. Rescoring function (e.g., GBVI/WSA dG).
    v. Number of poses retained per ligand.
  c. Run docking and export poses as: 06_Docking/<Protein>_<PDBID>_docking_poses.sdf (or MOE output format).
  d. Export docking scores table: 06_Docking/<Protein>_<PDBID>_docking_scores.csv.
  e. Repeat for each hub protein receptor and keep outputs separated by gene/PDBID.

***Note:*** The exact iteration number may be adjusted according to the desired conformational sampling depth and available computational resources.

26. Evaluate docking results and summarize the top-ranked poses.
  a. Rank poses by docking score and select the top-ranked pose for each receptor.
  b. Create a unified summary table across receptors: 06_Docking/docking_summary.csv with columns: Gene, PDBID, Pose_rank, Docking_score, Key_interacting_residues, Interaction_types (H-bond/hydrophobic/pi).
  c. Quality control: if the PDB contains a co-crystallized ligand, perform redocking of the native ligand and record RMSD (target < 2.0 Å). Save as 06_Docking/<Protein>_<PDBID>_redock_RMSD.txt.

***Note:*** Docking scores are used for relative ranking within the same docking setup. Lower scores generally indicate stronger predicted ligand–receptor affinity, but experimental validation is still required.

**Figure 8:**
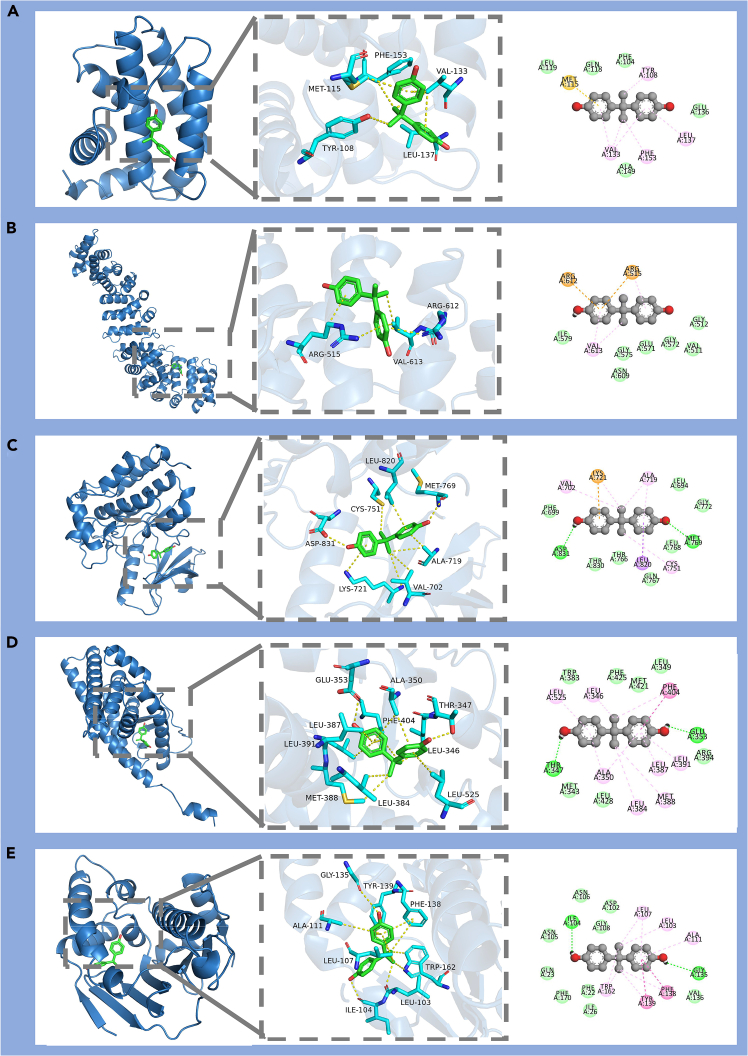
Molecular docking results of the selected environmental pollutant with the prioritized hub proteins Representative molecular docking results showing the binding of BPA to the five prioritized hub proteins identified from the network analysis. (A) BCL2. (B) CTNNB1. (C) EGFR. (D) ESR1. (E) HSP90AA1. For each target, the left panel shows the overall docking pose of BPA within the three-dimensional protein structure, the middle panel shows an enlarged view of the binding pocket and the surrounding interacting residues, and the right panel shows the corresponding two-dimensional interaction map. Across these targets, BPA was predicted to fit into the binding cavities and form multiple non-covalent interactions with neighboring amino acid residues, including hydrophobic contacts and hydrogen bond-related interactions. These docking results provide structure-level support for the potential binding of BPA to the prioritized targets and complement the upstream network and enrichment analyses.

***Note:*** In the present worked example, molecular docking and structural visualization were performed using MOE, ChemOffice (Chem3D), PyMOL, and Discovery Studio Visualizer. Appropriate free or open-source software (e.g., AutoDock Vina for docking and Avogadro for ligand preparation) may also be used, although exact numerical outputs may differ across platforms because of differences in docking algorithms and scoring functions.

27. Visualize the optimal docking poses and ligand–receptor interactions using PyMOL 3.1 and Discovery Studio Visualizer.
  a. Export the selected best pose complex from MOE as PDB: 06_Docking/<Protein>_<PDBID>_bestpose_complex.pdb.
  b. PyMOL visualization: load the complex, display ligand as sticks, highlight binding pocket residues (within 4 Å) and generate a publication image: 06_Docking/Figure 8_<Protein>_pose.png.
  c. Discovery Studio Visualizer: generate 2D interaction diagram (H-bonds, hydrophobic contacts) and save as: 06_Docking/Figure 8_<Protein>_2D_interactions.png.
  d. Merge all these panels together and save as: 06_Docking/Figure 8.pdf (Figure 8).

**CRITICAL:** Ensure that the docking grid or binding pocket is correctly defined around the active site of the receptor; incorrect grid placement may result in unreliable docking poses.

**Pause point:** Docking results and binding poses can be saved for downstream comparison with other ligands or further structural refinement.

## Expected outcomes

This protocol yields an integrated computational framework linking environmental pollutants to disease-associated molecular mechanisms. Users obtain pollutant- and disease-related target lists, construct compound–target–disease and PPI networks, and identify key hub proteins and enriched biological pathways. Molecular docking provides a structure-based plausibility assessment of key interactions. The expected outcome is a reproducible dataset that reveals potential molecular targets and mechanistic pathways through which pollutants may influence human health.

## Limitations

This protocol relies primarily on publicly available databases and computational prediction tools, which may introduce uncertainty due to incomplete or biased data. The accuracy of identified targets depends on the quality, curation, and update frequency of each database, and predicted interactions may include false positives or fail to capture context-specific targets. Network construction and enrichment analyses are also influenced by user-defined thresholds and filtering criteria, which may affect the identification of hub genes and enriched pathways. Importantly, this workflow does not incorporate temporal exposure information, dose-response relationships, or cell-type-specific resolution, all of which are important determinants of toxicological effects in biological systems.^22^ In addition, molecular docking provides only structure-based, theoretical support for ligand–receptor interactions and does not fully account for protein flexibility, dynamic conformational changes, pharmacokinetics, or *in vivo* biological complexity. Therefore, this workflow should be interpreted as a hypothesis-generating and prioritization framework that can guide downstream experimental validation, rather than as definitive evidence of causal toxicological mechanisms.

## Troubleshooting

### Problem 1

Inconsistent or incomplete retrieval of pollutant-related targets from databases (related to steps 1–4).

### Potential solution

Verify that the compound name and canonical SMILES are correctly entered in PubChem, ChEMBL, STITCH, and SwissTargetPrediction. Restrict the search to *Homo sapiens* and remove duplicates after merging results. If certain databases return no results, double-check the SMILES structure or use alternative chemical identifiers.

### Problem 2

STRING database returns too few or too many interactions (related to steps 11–12).

### Potential solution

Adjust the confidence score threshold. A higher cutoff (≥0.700) increases reliability but reduces node number; a lower cutoff (≤0.400) increases connectivity but may include false positives. Balance network density and interpretability before exporting data to Cytoscape.

### Problem 3

Cytoscape or CytoHubba plugin crashes or freezes during network analysis (related to steps 13–15).

### Potential solution

Verify sufficient system memory (≥16 GB recommended). Simplify the network by filtering weak edges or high-degree nodes. Reinstall Cytoscape and CytoHubba if instability persists, or perform centrality analysis in R using the igraph package.

### Problem 4

Enrichment analysis in R fails or produces empty output (related to steps 16–18).

### Potential solution

Confirm that all gene identifiers are standardized using UniProt and converted to official gene symbols. Ensure all required R packages (e.g., clusterProfiler, org.Hs.e.g.db) are installed and updated. If errors persist, clear the workspace and rerun the analysis with a reduced dataset.

### Problem 5

Molecular docking generates unrealistic poses or fails to converge (related to steps 19–27).

### Potential solution

Re-check receptor and ligand preparation in MOE to ensure proper protonation/tautomer states, removal of non-essential waters, ions, and co-crystallized ligands, and correct assignment of partial charges. Confirm that the binding site is correctly defined (e.g., by the co-crystallized ligand position or pocket detection) and that the docking region fully covers key residues. Increase the number of placements/poses and refinement iterations, and repeat docking using different initial placements. If convergence remains unsatisfactory, repeat docking using an alternative PDB structure with higher resolution or a ligand-bound conformation, and re-validate the protocol by redocking the native ligand to reproduce its experimental pose.

## Resource availability

### Lead contact

Requests for further information and resources should be directed to and will be fulfilled by the lead contact, Yanggang Hong (sun160414@icloud.com).

### Technical contact

Technical questions on executing this protocol should be directed to and will be answered by the technical contact, Yanggang Hong (sun160414@icloud.com).

### Materials availability

This study did not generate new unique reagents.

### Data and code availability

All data and scripts used in this protocol are available from the corresponding author upon reasonable request.

## Acknowledgments

The authors acknowledge the publicly available databases and software resources that made this analysis possible. The graphical abstract was created using BioRender.com. This work was supported by the 10.13039/501100013254National College Students Innovation and Entrepreneurship Training Program (No. 202510343017). The funder had no role in the study design, data collection, analysis, interpretation, or report writing.

## Author contributions

Y.H. conceptualized the protocol, performed the computational analyses, performed validation, wrote the original draft, reviewed and edited the manuscript, and supervised the project. J.L. reviewed and edited the manuscript. J.Z. performed validation and reviewed and edited the manuscript. D.W. performed validation and reviewed and edited the manuscript. F.C. performed validation. Y.M. reviewed and edited the manuscript. Y.W. reviewed and edited the manuscript.

## Declaration of interests

The authors declare no competing interests.
